# The Arterial Baroreflex Resets with Orthostasis

**DOI:** 10.3389/fphys.2012.00461

**Published:** 2012-12-07

**Authors:** Christopher E. Schwartz, Julian M. Stewart

**Affiliations:** ^1^Department of Physiology, The Center for Hypotension, New York Medical CollegeValhalla, NY, USA; ^2^Department of Pediatrics, The Center for Hypotension, New York Medical CollegeValhalla, NY, USA

**Keywords:** cardiovascular regulation, standing, sympathetic nerve activity, heart rate, ventilation

## Abstract

The arterial baroreflexes, located in the carotid sinus and along the arch of the aorta, are essential for the rapid short term autonomic regulation of blood pressure. In the past, they were believed to be inactivated during exercise because blood pressure, heart rate, and sympathetic activity were radically changed from their resting functional relationships with blood pressure. However, it was discovered that all relationships between carotid sinus pressure and either HR or sympathetic vasoconstriction maintained their curvilinear sigmoidal shape but were reset or shifted so as to best defend BP during exercise. To determine whether resetting also occurs during orthostasis, we examined the arterial baroreflexes measured supine and upright tilt. We studied the relationships between systolic BP and HR (the cardiovagal baroreflex), mean BP, and ventilation (the ventilatory baroreflex) and diastolic BP and sympathetic nerve activity (the sympathetic baroreflex). We accomplished these measurements by using the modified Oxford method in which BP was rapidly varied with bolus injections of sodium nitroprusside followed 1 min later by bolus injections of phenylephrine. Both the cardiovagal and ventilatory baroreflexes were “reset” with no change in gain or response range. In contrast, the sympathetic baroreflex was augmented as well as shifted causing an increase in peripheral resistance that improved the subjects’ defense against hypotension. This contrasts with findings during exercise in which peripheral resistance in active skeletal muscle is not increased. This difference is likely selective for exercising muscle and may represent the actions of functional sympatholysis by which exercise metabolites interfere with adrenergic vasoconstriction.

## Introduction

Standing up reduces venous return by translocating a large fraction of central blood volume, in excess of 500 ml in the adult human, to the dependent body parts. After mechanical equilibrium is re-established during continued standing, microvascular filtration from plasma to interstitium continues to reduce blood volume (Levick and Michel, 2010). Partial restitution of blood volume depends on lymphatic activity and reabsorption of interstitial fluid into the blood volume (Huxley and Scallan, 2011). Nevertheless, there is a net reduction in blood volume and venous return, and thus a net reduction in cardiac output (CO), cerebral blood flow, central blood volume, and stroke volume during quiet standing (Rowell, 1993). Total peripheral resistance (TPR), sympathetic nervous activity, and blood pressure are increased. Diastolic BP increases more than systolic blood pressure and the resultant decrease in pulse pressure coincides with the reduction in stroke volume when upright.

Orthostatic tolerance depends on intact intrinsic vascular structure and function, intact control of vasomotor function, adequate central blood volume and oxygen carrying capacity, and intact physical compensatory mechanisms including intact skeletal and respiratory muscle pumps (Wang et al., 1960; Miller et al., 2005). Rapid changes in blood pressure during orthostasis are primarily buffered by the arterial baroreflexes (Aviado and Schmidt, 1955; Mancia, 1975; Sanders et al., 1988) which comprise a negative feedback inhibitory stretch reflex (Sagawa, 1983). Therefore, a decrease in blood pressure reduces stretch of unmyelinated receptors in the carotid bifurcation and along the aortic arch causing a reflex increase in heart rate of primarily parasympathetic origin (Katona et al., 1970), and an increase in vasoconstriction of primarily sympathetic origin along with beta adrenergic mediated adrenal medullary secretion.

Increased arterial pressure during orthostasis should inhibit orthostatic sympathoexcitation which is instead increased (Burke et al., 1977). Prior investigations attribute this to a change in baroreflex sensitivity (Cooper and Hainsworth, 2001; Fu et al., 2006). Increased heart rate and adrenergic vasoconstriction are critical to maintaining blood pressure when upright and their absence is characteristic of autonomic failure causing neurogenic orthostatic hypotension (Freeman et al., 2011).

Changes in arterial baroreflexes have been found during exercise where it has been determined that the arterial blood pressure and heart rate stimulus-response derived from isolated carotid sinuses in dogs were displaced upward proportionate to exercise work load, with unchanged curve characteristics (Melcher and Donald, 1981); the same relationship was later seen in humans (Potts et al., 1993) where it was represents a resetting of the sigmoidal (Kent et al., 1972) cardiovagal and sympathetic baroreflex relations such that an upward and rightward shift of the stimulus-response (carotid sinus baroreflex pressure vs. heart rate, sympathetic nerve activity, arterial pressure) occurs (Raven et al., 1997; Fadel et al., 2001; Fadel and Raven, 2012). Subsequent investigations indicate that baroreflex resetting during exercise is a consequence of interactions of the arterial baroreflex with central command, cardiopulmonary pressure reflexes, and the exercise pressor reflex (Fadel and Raven, 2012).

The decrease in cardiopulmonary pressure that occurs with upright posture (Victor and Mark, 1985) led us to hypothesize that similar arterial baroreflex resetting occurred during orthostasis. We tested this hypothesis by examining the cardiovagal baroreflex (HR/RR-interval vs. systolic blood pressure), and the sympathetic baroreflex [MSNA vs. diastolic blood pressure (DBP)] during upright tilt. Our recent work also indicated the importance of a ventilatory efferent arm of the arterial baroreflex [ventilation vs. mean arterial pressure (MAP)] which we also examined. This was included because of the demonstrated relation between MAP and ventilation (Stewart et al., 2011), the known relation between orthostasis and increased ventilation (Matalon and Farhi, 1979), and the importance of increased ventilation and hypocapnia to diverse forms of orthostatic intolerance (Novak et al., 1998; Lagi et al., 2001; Stewart et al., 2006; Thijs and van Dijk, 2007).

## Materials and Methods

### Subjects

Ten healthy volunteer subjects aged 18–25 years (median = 21 years, five female). Average weight (± SD) was 74 ± 18 kg, average height was 173 ± 13 cm, average BMI was 22 ± 3 kg/m^2^. Subjects were free of all cardiorespiratory, autonomic, and systemic illnesses, took no medications and were non-smokers. There were no trained athletes. Informed consent was obtained. All protocols were approved by the Committee for the Protection of Human Subjects of New York Medical College.

### Instrumentation

Subjects refrained from eating for at least 8 h and had no caffeinated beverages for at least 24 h prior to testing.

Testing began at 9:30 a.m. Subjects were familiarized with the procedures used in the study prior to any instrumentation. An intravenous catheter was placed in the left antecubital vein. Peroneal microneurography was performed to measure muscle sympathetic nerve activity (MSNA). In brief, multiunit recordings of efferent postganglionic MSNA were obtained using the technique established by Vallbo et al. (1979). A tungsten microelectrode was inserted into a muscle fascicle of the peroneal nerve, posterior to the fibular head (Wallin et al., 1996) using a unipolar tungsten electrode (uninsulated tip diameter 1–5 μm, shaft diameter 200 μm; Frederick Haer & Co.). Nerve activity was amplified with a gain of 100,000, then band pass filtered (0.7–2 kHz), and integrated using a 0.1 s lag (University of Iowa, Iowa City). A low impedance reference electrode was inserted a 1–2-cms away. Stable MSNA recordings were pulse synchronous, had increased burst counts during stage II of the Valsalva maneuver and were insensitive to sudden clapping or stroking of the skin. Integrated MSNA appear as upright “bursts.” Identified bursts were expressed as burst count (bursts/min), and total MSNA (area under the curve for all bursts). Total MSNA was obtained after first normalizing each burst for any given subject by dividing all burst, supine, and upright by the largest burst amplitude during the baseline supine period and multiplying by 1,000. Total MSNA results were expressed as arbitrary units for each subject.

Subjects were instrumented for electrocardiogram (ECG), heart rate, and rhythm. Baseline beat to beat AP was collected while supine using finger photoplethysmography (Finometer, FMS, Amsterdam) calibrated against an oscillometric BP cuff. CO was estimated using the Finometer which models the circulation as an adaptive Windkessel and which was calibrated against an inert gas rebreathing apparatus (Innocor, Innovision, Denmark) using 0.1% sulfur hexafluoride (SF_6_). Respirations were monitored by pneumotachography (Hans Rudolph Inc., MO, USA) using a tight fitting face mask. The pneumotachograph was calibrated using a 3-L syringe. End tidal carbon dioxide (ETCO2) was measured with a capnograph using nasal prongs connected to a side stream capnograph while oxygen saturation was measured using standard pulse oximetry (Capnocheck^®^ Sleep Capnograph/Oximeter, Tri-Anim Health, and Sylmar, CA, USA). A chest impedance band (Respitrace, NIMS Inc., Miami, FL, USA) was also used to monitor relative respiratory excursions.

### Protocol

**Figure 1:**
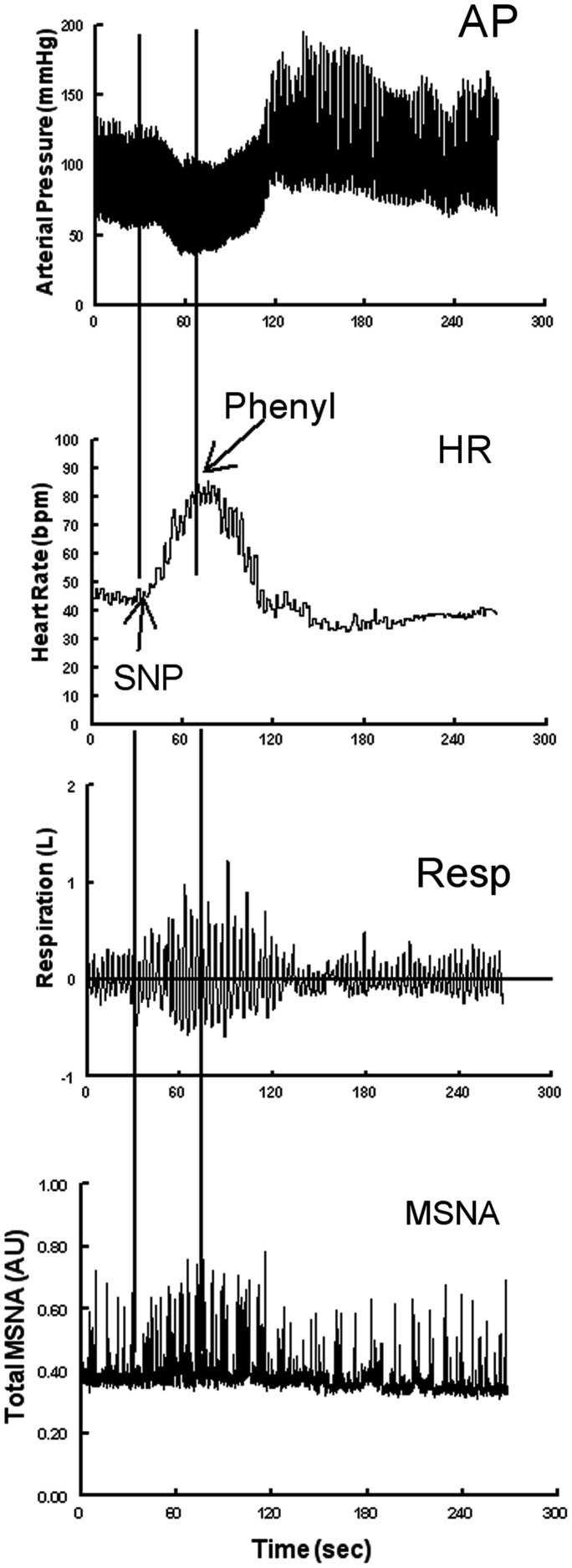
**Representative neurovascular and respiratory responses to the modified Oxford maneuver in which a sodium nitroprusside (SNP) bolus is followed 1 min later by phenylephrine**. From top to bottom the figure shows arterial pressure, heart rate, respirations, and MSNA bursts.

Following a 30 min acclimatization period, data were recorded throughout a 10 min supine baseline period. Baroreflex data were assessed using the modified Oxford method. An intravenous bolus injection of 100 μg sodium nitroprusside (SNP) was followed 1 min later by an intravenous injection of 150 μg phenylephrine (Figure 1). The method decreases AP by approximately 15–20 mmHg and subsequently increases AP by approximately 15–20 mmHg above baseline (Halliwill et al., 2003; Lipman et al., 2003). This results in an increase in heart rate (equivalently decrease in RR-interval), an increase in ventilation (Stewart et al., 2011), and an increase in sympathetic activity as shown in Figure 1.

Subjects were then allowed to recover and return to baseline over a 30 min period.

Following supine measurements subjects were tilted to 60° upright for 10 min. Heart rate, RR-interval, MSNA, respiratory, and AP data during minutes 1–5 were used for establishing a new upright baseline. The Oxford method was repeated at 5 min following the start of tilt tilt using the methods described above. After upright heart rate and AP returned to near pre-Oxford levels, subjects returned to the supine position. All subjects were able to tolerate the 10 min tilt and none reported symptoms of pre-syncope.

### Data analysis

Approximately 5 min of baseline “pre-Oxford” data were analyzed preceding performance of the Oxford method in the supine position. Also, upright baseline data were obtained during minutes 1–5 of HUT to avoid the fluid equilibration phase that occurs during the first minute of tilt (Wieling et al., 2007). Data were collected continuously during the hypotensive and hypertensive phases of the Oxford method in both supine and upright positions and included ECG, CO, systolic (SBP), and diastolic (DBP) blood pressure, and MAP calculated using the Finometer. TPR was computed using the formula TPR = MAP/CO (mmHg/L/min). RR-interval and heart rate were calculated from the EKG. MSNA bursts were detected by custom software that automated collection of bursts and burst counts with human operator oversight. All MSNA analysis was done by a single analyst. Data were sampled at 200 Hz using custom signal processing software and analyzed off-line.

### Cardiovagal baroreflex determined by the modified oxford method

Heart rate was plotted as a function of SBP to generate the cardiovagal baroreflex response during the modified Oxford method. The heart rate during each heart beat was calculated as 60,000/RR-interval (ms). Using all RR-intervals and SBP values from the onset of decrease in SBP until maximum SBP introduces “hysteresis” (Studinger et al., 2007); the same value of SBP during the decrease and increase of blood pressure can be associated with different RR-intervals. To avoid this complication we adopted the strategy of (Hunt and Farquhar, 2005) who restricted data acquisition to the rising arm of SBP (i.e., from the minimum until the maximum SBP was achieved).

### Baroreflex mediated sympathetic activation determined by the modified oxford method

Efferent baroreflex regulation of sympathetic activity was provided by the relationship between MSNA and DBP during the drug boluses. DBP correlates best with MSNA in humans (Sundlof and Wallin, 1978). MSNA bursts were time lagged by approximately 1.3 s from their respective triggering *R*-wave. Each burst was therefore shifted by the actual lag computed by our software. The shift needed to account for time lags varying from subject to subject.

A shifted and denoised file of bursts was created using normalized burst areas. Inter-burst activity was set to zero. Similarly, a file of burst counts was created such that each count comprised a discrete “delta” function at the peak of the burst with unit area corresponding to unit count at a given time. Thus, burst area per minute (total MSNA) or burst count per unit time could be simply obtained by taking the integral of burst area or burst count over the time in question.

### Ventilatory baroreflex determined by the modified oxford method

Efferent baroreflex regulation of ventilation was provided by the relationship between MSNA and MAP during the drug boluses. Respiratory volumetric changes were converted to expiratory minute volume (*V*_E_) using a differencing procedure by which the volume of air exhaled was calculated for each breath and divided by the total time in seconds of that breath, then multiplied by 60 (s/min). As calculated from raw pneumotachometer data, this equaled the integrated flow during expiration divided by the breath duration from onset of inspiration to offset of expiration (=onset of the next breath) multiplied by 60. MAPs corresponded best to *V*_E_. A time delay between MAP and ventilation would be expected based on neuromechanical issues. This was observed. Usually this varied between 0 and 3 s. Delays were related to the time to onset of the first breath following the start of decreasing blood pressure due to SNP used in the Oxford method. Delays were compensated by shifting respirations such that onset of change in respiration corresponded to onset of change in pressure. This is similar to practices used to align muscle sympathetic nerve recordings to corresponding RR-intervals (Delius et al., 1972).

Our data comprised pairs of time series indexed to the cardiac cycle: SBP and RR-interval, MAP and *V*_E_, DBP, and MSNA bursts or total MSNA as delta functions. In each, the pressure (SBP, MAP, or DBP) in mmHg corresponding to each cardiac cycle was assigned to the entire cycle in the time series. Similarly we assigned the magnitude of the heart rate in beats per min, *V*_E_ in L/min, and MSNA bursts per min or total MSNA to their corresponding cardiac cycle, thus creating HR, *V*_E_, MSNA, and pressure tachograms. A tachograms replaces a continuous set of values with its average for a given cardiac cycle. For a fiducial value such as diastole or systole, the tachygram assigns that value to the entire cardiac cycle. Thus, when the time series for a tachograms is closely inspected a series of steps of duration equal to the cardiac cycle and of amplitude equal to the measurement is obtained. For ventilation each step had only a portion of a respiratory cycle corresponding to it (within the particular cardiac cycle) or there could be overlap overlap with adjacent *V*_E_ steps. In either case a time average of the *V*_E_ falling within a particular cardiac cycle was performed. Thereafter, each cardiac cycle, pressure, and corresponding paired variable (HR, *V*_E_, MSNA) were collapsed to a discrete (point) value corresponding to a single discrete cardiac cycle. These aligned digital sequences of pressure and paired variables were then sorted on pressure using the Quicksort algorithm (Hoare, 1962) from smallest to largest. We next binned pressures into pressure bins of 1–2 mmHg depending on data. Several values of any given pressure sequence typically fell within a given bin and were averaged to obtain an averaged bin pressure. Similarly, corresponding points of the paired variable (RR-interval, *V*_E_, MSNA) point values were effectively binned to obtain an average of the paired over the specific bin. There were typically no empty bins because of the averaging procedure. We performed a weighted least squares fit of RR-interval against SBP where the weights were the number of points that fell into a specific bin divided by the total number of points in all bins.

Cardiovagal baroreflex curves (HR vs. SBP), ventilatory baroreflex curves (*V*_E_ vs. MAP), and sympathetic activity baroreflex curves (MSNA burst or total MSNA vs. DBP) were constructed for each subject while supine and during HUT by plotting pressure on the abscissa and the paired variable on the ordinate during the Oxford maneuver. A logistic (sigmoid) curve was fitted for each subject and each pressure pair during both positions using the Levenberg–Marquardt non-linear least squares algorithm (Marquardt, 1963) and gave a much better fit in the least squares sense compared to a straight line. An *r*^2^ of 0.85 or greater was obtained with each fit. The logistic curve was determined by four parameters *a*_0_, *a*_1_, *a*_2_, *a*_3_, and calculated using the following equation:

$$\text{Paired variable}=a_{0}+\frac{a_{1}}{1+\exp\left[\frac{-\left(\text{AP}-a_{2}\right)}{a_{3}}\right]}$$

where:

*a*_0_ is the lower asymptote or “threshold” of the fit,
*a*_0_ + *a*_1_ is the maximum asymptote or “saturation” of the fit,
*a*_2_ is the value of AP at maximum slope (centering point),
*a*_1_/(4*a_3_) determines the maximum slope or “sensitivity.”

**Figure 2:**
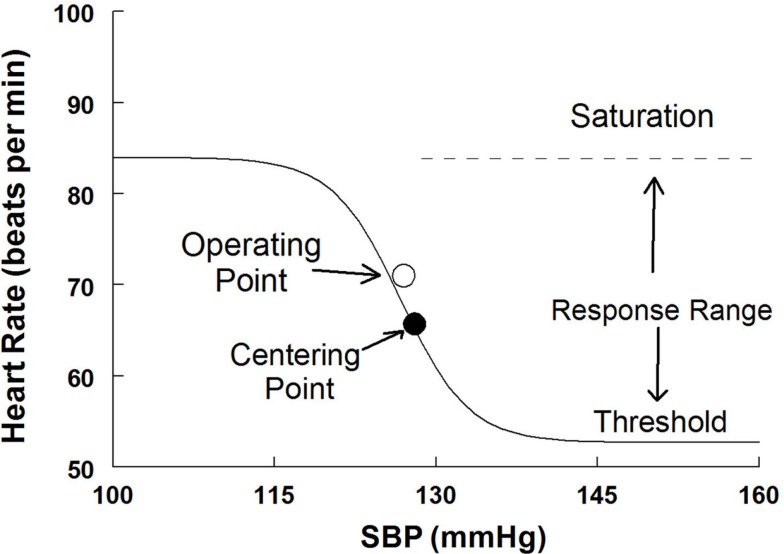
**A sigmoid fitted curve using the modified Oxford procedure to generate the HR vs. systolic blood pressure relation**. The operating point, centering point, threshold, saturation, and response range are illustrated.

Baroreflex sensitivity corresponds to the maximum slope (or maximum gain) of the generated sigmoid curve, while the operating point (OP) was determined by the measured pressure and its paired variable when supine or upright. A slope was also calculated at the centering point from the logistic fit equation. The baroreflex threshold was identified as the minimum asymptote of the sigmoid curve and the saturation was identified as the maximum asymptote of the curve. The response range was calculated as the difference between the threshold and saturation. An idealized curve is shown in Figure 2. Note that a least squares algorithm was used to fit individual curves over a range of Oxford-generated pressures and its paired variables. Therefore the OP for each curve did not in general fall on the fit curve as shown.

### Statistics

Because there were no discernible differences between the data from men and women, data from both groups were combined for analysis. Measurements made in the supine and HUT positions computed for each individual were compared using paired *t*-tests with a Bonferroni correction for multiple comparisons. Two-way analysis of variance for repeated measures was used for Oxford measurements comparing changes in response to SNP and phenylephrine in the supine and HUT positions. When appropriate, *post hoc* comparisons were performed using Tukey’s test. Differences were considered significant when *P *< 0.05. All values are reported as means ± SD. Results were calculated using SPSS (Statistical Package for the Social Sciences) software version 11.0.

## Results

### Effect of HUT

The HR increased with HUT (*P* < 0.001) while systolic, diastolic, and MAP did not change significantly. CO, and TPR also did not change significantly but there were significant increases in total MSNA and in MSNA counts (*P* < 0.01). Expiratory minute volume increased (*P* < 0.01) while respiratory rate failed to increase indicating an increase in tidal volume with HUT (Tables 1 and 2).

**Table 1:** **Supine and upright hemodynamics and MSNA pre-Oxford, after SNP bolus, and after phenylephrine**.

Measurement	Supine	HUT
**HR (bpm)**
Pre-Oxford	62 ± 4	86 ± 5^†^
SNP	94 ± 4*	121 ± 8*^†^
Phenylephrine	50 ± 3*	68 ± 4*^†^
**SBP (mmHg)**
Pre-Oxford	122 ± 5	125 ± 5
SNP	98 ± 7*	96 ± 5*
Phenylephrine	137 ± 5*	138 ± 7*
**DBP (mmHg)**
Pre-Oxford	67 ± 3	72 ± 2
SNP	50 ± 3*	46 ± 3*
Phenylephrine	78 ± 6*	83 ± 5*
**MAP (mmHg)**
Pre-Oxford	82 ± 2	87 ± 3
SNP	68 ± 4*	64 ± 5*
Phenylephrine	92 ± 2*	91 ± 4*
**CO (l/min)**
Pre-Oxford	4.8 ± 0.3	4.5 ± 0.4
SNP	6.8 ± 0.5*	6.4 ± 0.5*
Phenylephrine	3.6 ± 0.3*	3.7 ± 0.3*
**TPR (mmHg/l/min)**
Pre-Oxford	16 ± 2	17 ± 2
SNP	11 ± 1*	12 ± 1*
Phenylephrine	26 ± 3*	23 ± 3*
**Total MSNA (AU/min)**
Pre-Oxford	2112 ± 367	3153 ± 433^†^
SNP	7319 ± 656*	14677 ± 3018*^†^
Phenylephrine	1224 ± 311*	2023 ± 889*
**MSNA counts (Bursts/min)**
Pre-Oxford	17 ± 2	33 ± 6^†^
SNP	40 ± 4*	52 ± 6*^†^
Phenylephrine	11 ± 2*	29 ± 4*^†^

Values are mean ± SD. MSNA, muscle sympathetic nerve activity; HUT, head up tilt; SNP, sodium nitroprusside; SBP, systolic blood pressure; DBP, diastolic blood pressure; CO, cardiac output; TPR, total peripheral resistance; *P < 0.05 compared to pre-Oxford; ^†^*P* < 0.05 compared with supine.

**Table 2:** **Supine and upright respiratory data pre-Oxford, after SNP bolus, and after phenylephrine**.

Measurement	Supine	HUT
***V*_E_ (l/min)**
Pre-Oxford	7.8 ± 0.9	10.2 ± 1.0^†^
SNP	22.2 ± 7.1*	25.9 ± 7.8*
Phenylephrine	4.8 ± 0.6*	6.9 ± 0.8*^†^
**Respiratory rate (breath/min)**
Pre-Oxford	13.6 ± 1.1	13.3 ± 1.6
SNP	12.3 ± 1.2	13.2 ± 1.6
Phenylephrine	13.1 ± 1.5	12.7 ± 1.6
**ETCO_2_ (Torr)**
Pre-Oxford	43 ± 1	42 ± 1
SNP	38 ± 1*	37 ± 1*
Phenylephrine	44 ± 1	44 ± 1

Values are mean ± SEM. HUT, head up tilt; SNP, sodium nitroprusside; *V*_E_, expiratory minute volume; ETCO_2_, end tidal CO_2_; *P < 0.05 compared to Pre-Oxford; ^†^*P* < 0.05 compared with supine.

### Effect of the modified oxford maneuver on hemodynamics and MSNA, supine and upright

HR decreased with SNP (*P* < 0.001) and increased with phenylephrine (*P* < 0.001). Systolic, diastolic, and mean pressures decreased significantly (*P* < 0.001) with SNP and increased with phenylephrine. There were no differences when upright. In contrast CO increased with SNP and decreased with phenylephrine (*P* < 0.01) while TPR decreased with SNP and then increased with phenylephrine (*P* < 0.01). There were also no differences when upright. MSNA bursts and total MSNA increased with SNP and decreased to below baseline with phenylephrine. MSNA was significantly (*P* < 0.01) larger upright compared to supine (Table 1).

### Effect of the modified oxford maneuver on respiratory data

The respiratory rate was unaffected by SNP and phenylephrine either supine or upright. However, expiratory minute volume *V*_E_ increased with HUT (*P* < 0.05). The expiratory minute volume *V*_E_ also increased markedly with SNP and decreased with phenylephrine (*P* < 0.001) implying a large increase in tidal volume. ETCO_2_ was less affected with a smaller but significant (*P* < 0.01) fall in ETCO_2_ as the result of short lived hyperpnea during SNP; this failed to empty carbon dioxide reserves (Table 2).

Baroreflex curves were fit for each cardiopulmonary parameter for each subject, while supine and upright using sigmoidal curvilinear fits as stated above. Goodness of fit using a squared correlation measure (*r*^2^) varied from 0.80 to 0.98 with an average for each individual parameter of approximately of 0.90. For comparison linear fits resulted in squared correlation ranging from 0.4 to 0.8.

#### Cardiovagal baroreflex results supine and upright

**Figure 3:**
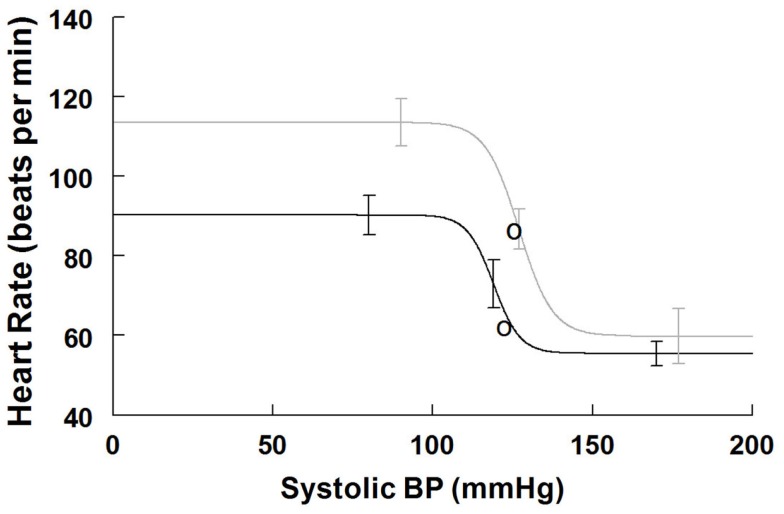
**Supine and upright cardiovagal baroreflex relationships were obtained by averaging data-fits over all subjects during the Oxford maneuver**. The upright curve is shifted upwards and to the right from supine but the maximum slope (sensitivity) is unaffected by posture.

Maximum cardiovagal baroreflex slope (sensitivity) is shown in Table 3. The slope is not statistically different supine or upright. The HR at maximum slope is significantly increased by HUT as are the threshold and saturation of the sigmoid curve. The response range is not different. Systolic BP at maximum gain is significantly increased by HUT (*P* < 0.01). Figure 3 shows supine and upright curves created from the averaged values of Table 3. OPs averaged over all subjects are shown. Since response range and slope are unchanged, the curves can be created by translating the centering point to an increased heart rate thereby resetting the relationship without changing its sensitivity as shown in Figure 3.

**Table 3:** **Calculated cardiovagal baroreflex sensitivity (slopes), threshold, saturation, and response range using the modified Oxford method**.

Calculated logistic parameters	Supine	HUT
Slope (sensitivity, max gain, bpm/mmHg)	−2.7 ± 0.7	−3.4 ± 1.0
SBP at maximum gain (mmHg)	119 ± 5	127 ± 3^†^
HR at maximum gain (bpm)	73 ± 6	87 ± 5^†^
Threshold (bpm)	55 ± 3	66 ± 7^†^
Saturation (bpm)	91 ± 5	106 ± 6^†^
Response range (bpm)	36 ± 5	44 ± 7

*Values are mean ± SD. bpm, Beats per minute; HUT, head up tilt; SBP, systolic blood pressure; ^†^*P* < 0.05 compared to the supine*.

#### Ventilatory baroreflex results supine and upright

**Figure 4:**
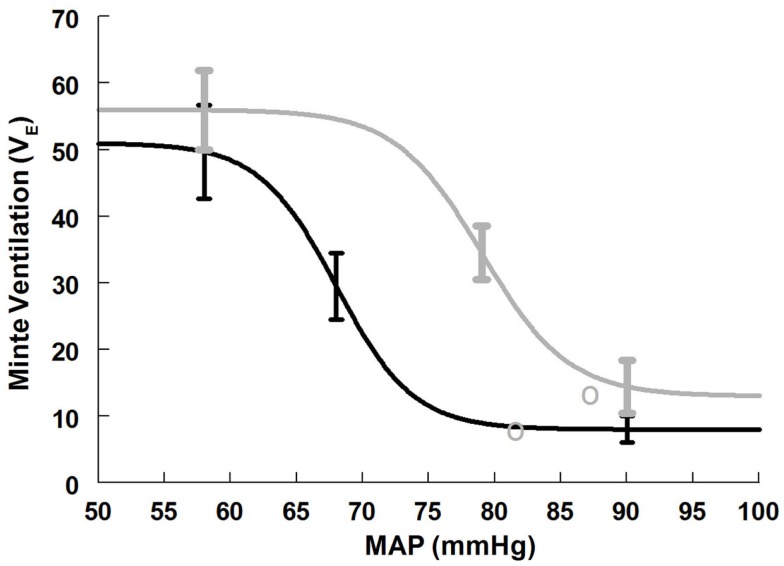
**Supine and upright ventilatory baroreflex relationships were obtained by averaging data-fits over all subjects during the Oxford maneuver**. The upright curve is shifted to the right from supine but the maximum slope (sensitivity) is unaffected by posture.

Maximum ventilatory baroreflex slope (sensitivity) is shown in Table 4. The supine and upright maximum slopes are not significantly different. The expiratory minute volume, *V*_E_, at maximum slope is not significantly increased during HUT nor are the threshold and saturation of the sigmoid curve. Thus, the response range is not different. Figure 4 depicts supine and upright curves created from the averaged values of Table 4. OPs averaged over all subjects are shown. Since response range and slope are unchanged, the curves can be created by translating the centering point to an increased *V*_E_, thereby resetting the relationship without changing its sensitivity as shown in Figure 4.

**Table 4:** **Calculated ventilatory baroreflex (slopes), threshold, saturation, and response range using the modified Oxford method**.

Calculated logistic parameters	Supine	HUT
Slope (sensitivity, max gain, ms/mmHg)	−3.7 ± 0.7	−3.3 ± 0.8
MAP at maximum gain (mmHg)	68 ± 3	79 ± 3^†^
*V*_E_ at maximum gain (l/min)	31 ± 5	35 ± 4
Threshold (l/min)	8 ± 2	13 ± 4
Saturation (l/min)	51 ± 7	56 ± 6
Response range (l/min)	41 ± 8	41 ± 6

*Values are mean ± SD. HUT, head up tilt; MAP, mean arterial pressure; *V*_E_, expiratory, minute ventilation; ^†^*P* < 0.05 compared to the supine*.

#### Total MSNA baroreflex response supine and upright

**Figure 5:**
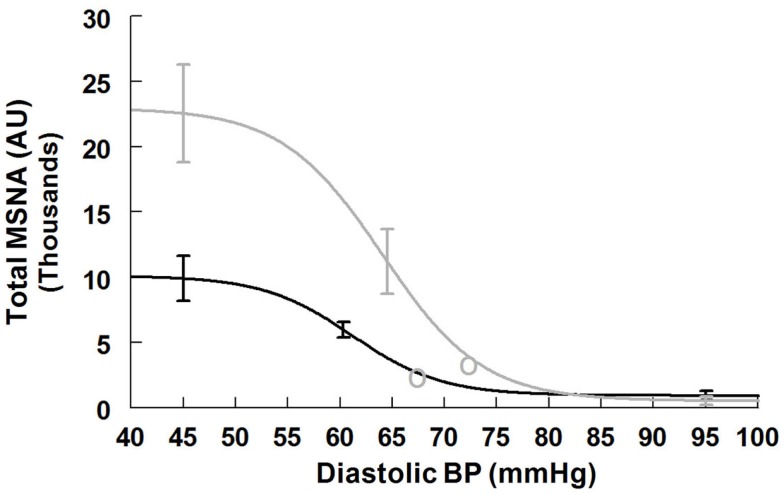
**Supine and upright total MSNA response relationships obtained by averaging data-fits over all subjects during the Oxford maneuver**. The upright curve is shifted from supine but the maximum slope (sensitivity), response range, and saturation are also increased when upright.

Maximum total MSNA baroreflex slope (sensitivity) is shown in Table 5. The slope magnitude is significantly greater upright compared to supine (*P* < 0.05). There is no difference in threshold but the centering point is shifted to the right and upwards (*P* < 0.01), and both upright saturation and response range are twice as large as corresponding supine quantities (*P* < 0.01). Figure 5 depicts supine and upright curves created from the averaged values of Table 5. Since both response range and slope are increased during HUT, the curves are not related by a simple translation of the centering point. Resetting in the sense of an unchanged shape of the sigmoid curve does not occur in the context of sympathetic activation during HUT as shown in Figure 5.

**Table 5:** **Calculated total MSNA baroreflex sensitivity (slopes), threshold, saturation, and response range using the modified Oxford method**.

Calculated logistic parameters	Supine	HUT
Slope (sensitivity, max gain, AU/mmHg)	−871 ± 167	−1084 ± 220^†^
DBP at maximum gain (mmHg)	60 ± 3	64 ± 3^†^
MSNA at maximum gain (AU/min)	5503 ± 582	11722 ± 2466^†^
Threshold (AU/min)	917 ± 282	698 ± 223
Saturation (AU/min)	10,080 ± 1871	22877 ± 3806^†^
Response range (AU/min)	9173 ± 1329	21457 ± 4306^†^

*Values are mean ± SD. HUT, head up tilt; SBP, systolic blood pressure; ^†^*P* < 0.05 compared to the supine*.

#### MSNA Count baroreflex response supine and upright

**Figure 6:**
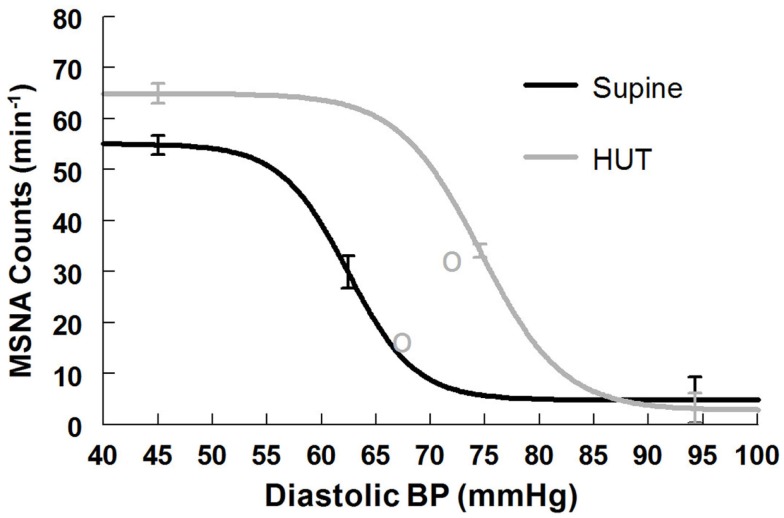
**Supine and upright MSNA burst count response relationships obtained by averaging data-fits over all subjects during the Oxford maneuver**. The upright curve is shifted from supine but the maximum slope (sensitivity), response range, and saturation are also increased when upright.

Maximum MSNA burst count slope (sensitivity) is shown in Table 6. The slope magnitude is significantly greater upright compared to supine (*P* < 0.05). There is no difference in threshold but the centering point is shifted to the right (*P* < 0.01). Upright saturation and response range are larger than corresponding supine quantities (*P* < 0.025) but difference in MSNA counts are far less dramatic compared to differences in total MSNA. Figure 6 depicts supine and upright curves created from the averaged values of Table 5. Although response range and slope are still increased during HUT compared to supine this difference is relatively reduced compared to differences in total MSNA. The curves cannot be created by simply translating the centering point. Resetting in the context of sympathetic activity augments the response to changing diastolic pressure as shown in Figure 6.

**Table 6:** **Calculated MSNA counts**.

Calculated logistic parameters	Supine	HUT
Slope (sensitivity, max gain, counts/mmHg)	−4.1 ± 0.9	−5.3 ± 1.0^†^
DBP at maximum gain (mmHg)	62 ± 3	74 ± 3^†^
MSNA at maximum gain (counts/min)	30 ± 3	34 ± 1^†^
Threshold (counts/min)	5 ± 2	3 ± 2
Saturation (counts/min)	55 ± 4	65 ± 3^†^
Response range (counts/min)	50 ± 5	62 ± 3^†^

*Values are mean ± SD. HUT, head up tilt; DBP, diastolic blood pressure; ^†^*P* < 0.05 compared to the supine*.

## Discussion

On the one hand our results show cardiovagal and ventilatory baroreflex resetting when upright compared to supine. HR and expiratory minute volume increase when upright and respond similarly to a change of blood pressure whether supine or upright. On the other hand, the sympathetic baroreflex response is increased overall when upright compared to supine. The baroreflex function curves that are generated by the modified Oxford maneuver are displaced with respect to their centering points but are also changed in shape and thus in sensitivity and response range.

### Resetting of the cardiovagal arterial baroreflex during orthostasis

The resetting of the cardiovagal baroreflex can be understood in terms of the vagal withdrawal that occurs with upright posture. This resetting is similar to findings during exercise because similar vagal withdrawal occurs in both. Moreover, the increase of heart rate with upright posture does not normally exceed the capabilities of cardiac parasympathetic withdrawal to increase heart rate (Brouha et al., 1939). The OP of the upright cardiovagal baroreflex is at a higher heart rate and has shifted into the more linear portion of the sigmoid HR-baroreflex relation. A similar shifting occurs during exercise. The change in cardiovagal baroreflex centering or set point results from the reduction of central venous pressure on standing which unloads the cardiopulmonary baroreflexes (Pawelczyk and Raven, 1989). Stroke volume is markedly reduced during standing or HUT (Wang et al., 1960; Harms et al., 1999). Since CO = SV × HR, the CO is less affected than stroke volume because heart rate increases when upright. Therefore the shift in the OP of the cardiovagal reflex and the resetting of the baroreflex curve favors and protects compensatory tachycardia without which chronotropic incompetence and orthostatic intolerance would supervene (Kawasaki et al., 2010).

### Resetting of the ventilatory arterial baroreflex during orthostasis

The resetting of ventilatory baroreflex is more difficult to explain since mechanisms remain hypothetical. Among potential mechanisms is the sympathoexcitation that occurs with orthostatic stress that can directly result in hyperventilation (Folgering, 1999). In this regard, large animal research indicates that projections from the arterial baroreceptors directly modulate respiratory activity (Gabriel and Seller, 1969; Richter and Seller, 1975), while sympathetic activation and vagal withdrawal stimulate peripheral and central chemoreflex activity (Heistad et al., 1975; Mancia, 1975).

Along with cardiovagal baroreflex resetting, ventilatory baroreflex resetting would protect CO by shifting the OP to a more linear portion of the *V*_E_ vs. MAP baroreflex curve when upright so that ventilation is enhanced. Hypotension results in increasing hyperpnea. The respiratory pump is engaged and is particularly effective in enhancing venous return to the heart during conditions of increased tidal volume (i.e., hyperpnea) by its effects on abdominal pressure, splanchnic emptying and femoral venous return (Takata et al., 1990).

### Resetting of the sympathetic arterial baroreflex during orthostasis

Our data indicate that the total MSNA – DBP relation is not similarly reset as are heart rate and ventilation. The upright curve is not simply a translated version of the supine curve. Rather it is potentiated by upright posture so that gain, saturation and response range are increased. Increased MSNA counts during standing has been previously reported (Burke et al., 1977) although linear data fits were used; it is unclear whether there could be enhanced unloading of the central compartment without any change in the functional relationship between blood pressure and MSNA (movement along a baroreflex functional curve) or a change in the functional relationship as we now report. Significantly increased total MSNA and burst count have been reported (Fu et al., 2004). During exercise, MSNA and leg vascular resistance appear to be dissociated (Shoemaker et al., 2000) even though similar increases in MSNA occur (Norton et al., 1999; Fadel et al., 2001; Ogoh et al., 2009). On the other hand increased MSNA also occurs with orthostasis but results in increased local peripheral resistance (Cooper and Hainsworth, 2001) which is sustained and correlates well with MSNA (Fu et al., 2006).

Thus, during orthostasis there is a functional relationship of MSNA with peripheral resistance; during exercise there is no well-defined relationship. This observation indicates certain fundamental differences in vascular regulation during exercise and orthostasis. Both stressors require adaptation of blood pressure control that includes resetting of the arterial baroreflexes; both maintain blood pressure stable or increased and defend against hypotension; and both produce generalized sympathetic activation comprising different strategies for dealing with these different vascular challenges. In dynamic exercise, vasodilation of actively exercising muscle occurs in response to metabolic and flow mediated factors promoting a large increase in active muscle blood flow. Increased blood flow depends on the decrease in peripheral resistance, on the actions of the skeletal and respiratory muscle pumps to enhance venous return to the heart, and on increased CO which is ensured by sympathetically mediated increase in cardiac contractility, by the Starling mechanism, and by cardiac afterload reduction (Kimball et al., 1993). Sympathetic vasoconstriction, in part mediated by the arterial baroreflex regulation, in part by central command, and in part by somatic reflexes such as the exercise pressor reflex (Donald et al., 1970), still occurs and would tend to counteract excessive skeletal muscle metabolic vasodilation. Local vasodilation and flow augmentation therefore reach a balance with sympathetic vasoconstriction. In addition, evidence suggests that sympathetic vasoconstrictive capability in actively exercising muscle is reduced in potency. Thus, for example, sympathetically denervated limbs may have similar vasodilation to exercise as sympathetically intact limbs (Beaconsfield, 1954). Also, functional sympatholysis – the blunting of vascular smooth muscle responsiveness to sympathetic vasoconstriction by metabolic factors in exercising muscle – can reduce MSNA-vascular smooth muscle transduction (Tschakovsky et al., 2002).

In orthostasis there is no sympatholysis, there is no pressor reflex, but there is central command evidenced by an increase in HR and BP in the seconds prior to standing or upright tilt. Standing up profoundly decreases venous return by almost instantly translocating a large fraction of central blood volume, in excess of 500 ml in the adult human, to the dependent body parts (Blomqvist and Stone, 1983). Because of this, until reflex vasoconstriction is established, there is a substantial transient decrease in blood pressure referred to as “initial orthostatic hypotension” (Wieling et al., 2007). Even with normal autonomic activation there is a decrease of CO by approximately 20%. Because there is no sympatholysis, transduction of MSNA to peripheral vasoconstriction occurs in monotonic fashion to provide a steady increase of sympathetic activity and adrenergic vasoconstriction with orthostatic stress. That is precisely what happens during upright stance. Sympathetic baroreflex curves show that when supine, both total MSNA and burst counts operate near the centering point where sensitivity is at maximum. With orthostasis, sympathetic sensitivity (gain) increases, and the OP for total MSNA shifts so as to remain near the centering point of the new upright shifted and augmented sigmoid curve. Burst counts appear to be close to their maximum which may best afford the overall increase in total MSNA. Thus, any decrease in blood pressure is prevented by a compensatory increase in MSNA operating over the optimum linear range while upright. This fits well with teleology: sympathetic activity is diminished when it is an impediment and enhanced when it is an advantage toward blood pressure maintenance. In the absence of effective sympathetic vasoconstriction, neurogenic orthostatic hypotension supervenes within seconds to a few minutes and syncope follows (Freeman et al., 2011).

### Limitations

A modicum of caution is recommended because MSNA measured by peroneal microneurography assesses sympathetic nerve activity at a single location. However, it is likely that sympathetic activity is widely increased during orthostasis. Thus, for example, vascular resistance is typically increased in the splanchnic and renal circulations. On the other hand, sympathetic vasoconstriction is generally blunted in the coronary and cerebral circulations due to autoregulation.

In addition the data obtained has not been compared to data from other experiments in which baroreflex function was assessed by lower body negative pressure (LBNP; el Bedawi and Hainsworth, 1994; Goldstein et al., 2002), although some LBNP data are used for qualitative comparison (Cooper and Hainsworth, 2001; Carter et al., 2009).

An additional limitation of the data is that the testing was not standardized to one particular phase of the menstrual cycle. Baroreflex function may be influenced by menstrual phase (Carter et al., 2009). However, there remains controversy with other investigators finding no effect of menstrual phase on baroreflex (Fu et al., 2009).

Performing only one modified Oxford trial rather than two or more averaged trials is a potential limitation because of the inherent variability in modified Oxford trials within a subject. However, this was necessitated by the time limits during upright tilt testing.

Finally, averaged OPs do not fall on averaged baroreflex curves. These are fit curves and non-linear least squares methods can introduce more error than linear methods. However, the fact that least squared fitting was used practically assures that the fitted curves do not go precisely through the resting OP but instead represent averages in a least squares sense of all points generated by the Oxford maneuver. On the other hand an average of the OPs is arithmetic (sum/number of values) which yields a somewhat different value.

### In summary

Arterial baroreflexes remain effective in the regulation and maintenance of blood pressure during orthostasis because the relationships between arterial blood pressure, heart rate, respiration, and sympathetic nerve activity shift or reset when upright. These shifts prevent hypotension through sympathetic activation and improve CO by an increase in heart rate and engagement of the respiratory-abdominal pump.

## Conflict of Interest Statement

The authors declare that the research was conducted in the absence of any commercial or financial relationships that could be construed as a potential conflict of interest.
